# Can Music Therapy Improve Cognition in Dementia as Measured with Magnetoencephalography: A Hypothesis Study

**DOI:** 10.3390/biomedicines14020452

**Published:** 2026-02-17

**Authors:** Benjamin Slade, Benedict Williams, Romy Engelbrecht, Will Woods, Sunil Bhar, Joseph Ciorciari

**Affiliations:** 1 Centre for Mental Health and Brain Science, Swinburne University of Technology, John Street, Hawthorn, Melbourne, VIC 3122, Australia; jciorciari@swin.edu.au; 2 School of Health Sciences, Swinburne University of Technology, John Street, Hawthorn, Melbourne, VIC 3122, Australia; 3 Independent Researcher, Melbourne, VIC 3122, Australia; 4 Department of Psychological Sciences, Swinburne University of Technology, John Street, Hawthorn, Melbourne, VIC 3122, Australia

**Keywords:** music therapy, neural networks, dementia, working memory, health intervention, compensation, magnetoencephalography

## Abstract

**Background/Objectives:** The incidence of dementia and the concurrent burden on healthcare will increase with a population that continues to age. Pharmaceutical interventions for dementia carry negative side effects, ineffectively treat underlying causes, and fail to prevent disease onset. Therefore, non-pharmaceutical interventions such as music therapy should to be explored as a standalone or co-therapy for dementia. Music therapy improves cognitive symptoms of dementia; however, the neural mechanisms underpinning these improvements are not fully understood. **Methods:** To investigate potential neural mechanisms, six participants with dementia completed the Standardised Mini Mental State Examination, an n-back task, and magnetoencephalography (MEG) scanning before and after a music therapy program structured around improving executive functioning. **Results:** After music therapy, scores on an n-back task improved, and the MEG data revealed increased connectivity in neural networks and areas associated with compensation during executive functioning tasks. Connectivity results suggest there is preliminary evidence that music therapy improves cognitive symptoms of dementia by activating compensatory neural networks and areas; however, given the small sample size, these results should be interpreted with caution. **Conclusions:** The results of this hypotheses study present music therapy as a potentially viable short-term intervention which may operate by targeting compensatory neural networks and could be a long-term intervention that incorporates positive modifiable lifestyle factors, protecting the brain from dementia.

## 1. Introduction

The incidence of dementia increases as the global population ages [1]. The estimated incidence of dementia globally in 2019 was 57.4 million people, and this will be increasing to 152.8 million people by 2050 [2]. The incidence of dementia is predicted to double every five years [3], with the global financial burden estimated to reach US$ 1.3 trillion in 2019 [4].

As there is no cure for dementia, addressing the incidence and financial burden is difficult. Pharmaceutical interventions are first-line approaches that manage symptoms only and have side effects [5]. Therefore, non-pharmaceutical interventions, such as working memory-focused activities, playing music, problem solving, and physical activity, may reduce the incidence of dementia [6] and relieve symptoms and therefore should be explored as therapies or co-therapies for dementia [1].

Music therapy is one such non-pharmaceutical intervention that is cost-effective, easily implemented, and can be designed to include working memory focused and physical activities. Symptoms of dementia such as depression, anxiety, and cognitive impairments improve in response to music therapy [7,8]. How music therapy mitigates these symptoms of dementia is not fully understood but likely involves the recruitment of neural resources and compensation in response to increased cognitive demands that are governed by functional neural networks [9].

Functional neural networks in dementia show continual dysfunctional change in response to increasing neuropathology [10]. This dysfunction begins in mild cognitive impairment (MCI), as neural networks show inter-network synchronisation [11]; in response, compensatory neural activity increases to maintain higher cortical functions. Increases in inter-network compensation has been shown to occur within the fronto-parietal control network (FPCN) and within the salience network (SN) to compensate for decreases in inter-network activation of the default mode network (DMN; [12,13]). Integrity of these three large-scale functional neural networks decreases with increasing dementia-related neuropathology, leading to the cognitive and behavioural impairments characteristic of dementia (e.g., agitation and episodic and working memory decline [14,15]).

Targeting compensatory activity in neural networks that occurs in response to non-pharmaceutical interventions is shown to improve working memory in MCI [16]. There is little research investigating whether music therapy can increase neural network activity to improve cognitive functioning in dementia, despite music therapy engaging cognitive functions such as conflict monitoring, inhibitory control, and working memory [17], cognitive functions supported and governed by the FPCN [18,19]. As the FCPN shows neural compensatory activity in older adults and mild AD [20], music therapy could be used to enhance activation of these large-scale networks to improve and maintain cognitive functions for people with dementia.

### 1.1. Executive Functions, Neural Networks, and Dementia

Executive functioning processes (e.g., working memory) are shared across a number of brain areas including the parietal, sensory, temporal, and prefrontal cortices [21]. Executive functioning processes specifically rely on communication within and between three large-scale brain networks—FPCN, SN, and the DMN—to perceive and process sensory information to support and maintain executive functioning [21,22].

Dementia neuropathology often leads to impaired executive functioning, working memory, and attentional control systems [23], specifically for activities such as holding and manipulating auditory and visual information [24], carrying out instructions, processing sequential stimuli, switching tasks, and when continuous attention tasks demand different cognitive loads [23]. These working memory and executive function impairments are linked with dysfunctional and disorganised activity of FPCN, SN, and the DMN [21].

When completing working memory tasks, neural resources are diverted from the DMN to task-relevant processes and neural networks, leading to deactivation of the DMN [25,26]. As working memory load increases, the rate of DMN deactivation slows in healthy older people when compared to healthy adults, suggesting possible compensatory activity [26]. When the DMN fails to deactivate in healthy older adults during cognitively demanding tasks, this often reflects lapses in attention and would then lead to the FPCN and the SN increasing their compensatory activity [27].

The FPCN prioritises and coordinates information flow across multiple networks, including the DMN [28], and transfers task components such as task rules, knowledge, and skills from previous tasks to novel tasks [18]. The FPCN is a core memory network for working memory, as it is involved in task switching and maintenance, goal-directed behaviour, and also coping with non-routine task demands [29]. The FPCN becomes dysfunctional in dementia during working memory tasks as decreased activity occurs in the frontal areas while activation of the posterior parietal cortex increases [30,31]. This activation pattern during working memory tasks occurs in response to dysfunctional patterns of the FPCN and SN as the brain engages alternative pathways (e.g., inferior partial lobule, the medial frontal gyrus, and the left inferior frontal gyrus) to maintain cognitive performance [20].

The SN is a large-scale neural network that flexibly controls aspects of goal-directed behaviour. When salient stimuli are detected during executive functioning tasks, the SN engages the FPCN [32,33,34]; however, in dementia, this is disrupted, as the SN becomes dysfunctional with progressing neuropathology. This is partly driven by decreased activity within and between the SN and the Locus Coeruleus (LC; [35,36]), resulting in weaker signalling to activate the FPCN and deactivate the DMN [22]. This disruption leads to dysfunctional bottom-up processing of salient stimuli and communication between the FPCN and DMN depleting cognitive resources, increasing cognitive fatigue [22], and disrupting maintenance of working memory and executive functions [37].

### 1.2. Improving Working Memory and Executive Functioning Performance in Dementia

Improvements to working memory from cognitive interventions that target the FPCN suggest this is occurring with cognitive reserve [38]. When the FPCN is activated, additional neural resources are recruited to a task [39], or compensatory activity in the FPCN increases to maintain cognitive functioning [40]. Increased neural compensation within the FPCN is shown in dementia participants across working memory, executive functioning, attention, and episodic memory domains [14,15]. This wide-spread dysfunction may result in the recruitment of other neural areas that are not directly associated with the task or function of the network to maintain cognitive performance [41].

The FPCN is consistently activated when completing the n-back task [42], as the task involves working memory and executive function (attending to consistent stimuli, holding information in memory, updating stored information, and inhibiting responses; [43]), while specific areas of the FPCN support different components of the n-back. For example, the dorsolateral prefrontal cortex (DLPFC) maintains stimuli in memory, and activation of the posterior parietal cortex occurs when manipulating information and switching attention [44].

People with dementia react more slowly and are less accurate when responding to target and non-target 0, 1, and 2 n-back conditions compared to healthy aged-matched and MCI participants [45,46,47]. Lapses in attention on trial-by-trial cognitive tasks, like the n-back task, are common in dementia and are associated with the inability of the DMN to deactivate and increased activation of the posterior cingulate cortex (PCC), the medial frontal gyrus, and the anterior cingulate [48,49]. As the PCC is the hub of the DMN, the increased activation may be compensatory, recruiting additional attentional networks such as the FPCN and SN to perform trial-by-trial tasks [48].

Performance on the n-back, and similar tasks, can be improved with training or non-pharmaceutical interventions that target the neural networks responsible for the cognitive impairment [16,50]. Cognitive interventions used with the intention of improve symptoms of dementia, such as encoding and recall, problem solving, conversation skills, visuospatial tasks, creative activities, and task repetition, have shown moderate to large effect sizes [51], and transfer effects are observed to other cognitive abilities unrelated to music [52] for older adults [53].

The FPCN, the SN, and the DMN are implicated in working memory and executive functioning, and their functions are impacted by dementia. As music therapy involves cognitive intervention with a moderate to high effect on improving dementia symptoms, improving the cognitive functions these large-scale neural networks govern, and showing improved scores on the Mini Mental State Examination (MMSE [54,55]), it is therefore worthwhile investigating whether music therapy can change the inter- and intra-network communication to improve cognitive functioning in dementia.

### 1.3. Applying Music Therapy to Improve Working Memory and Executive Functioning in Dementia

Music therapy is a useful therapy or co-therapy for improving working memory in dementia. Empirical evidence shows that participants with AD recall more novel lyrics than novel words [56], can (when prompted) sing to and recognise melody compared to spoken words [57], and, when sung, can recall more novel songs compared to the same songs when spoken [58]. This is further supported by Satoh et al. [59], showing that after a 6-month karaoke training program with dementia participants, improvements were observed in psychomotor speed and dementia symptoms. These improvements were then found to be associated with decreased activation of the angular gyrus and the left lingual gyrus [59], suggesting musical training facilitates different cognitive strategies and can alter the organisation and efficiency of neural activation in dementia [59].

Music is perceived across many neural areas in the healthy brain, including the temporal lobes and hippocampus [60]. People with dementia can still perceive, engage with, and reminisce to music despite temporal lobe and hippocampal dysfunction and atrophy [61], suggesting different neural pathways and areas perceived [58] and which is further evidenced by effective music-based reminiscence therapy for people with dementia [62,63]. Specifically, the pre-supplementary motor area and anterior cingulate gyrus (ACG) are shown to be critical for music perception and storing musical information in long-term memory, and these areas are preserved in dementia. Furthermore, the pre-supplementary area and the ACG show less neural atrophy and deposits of Amyloid Beta compared to areas of the temporal lobe [61]. This evidence suggests that the ACG connects to the pre-supplementary motor area when responding to stimuli [33], and as the functional hub of the SN [22,34], music therapy programs may be able to be designed to target the ACG and to increase the activation of the SN, therefore increasing activation of the FPCN and improving working memory and executive functioning [40].

### 1.4. Present Study

**Figure 1:**
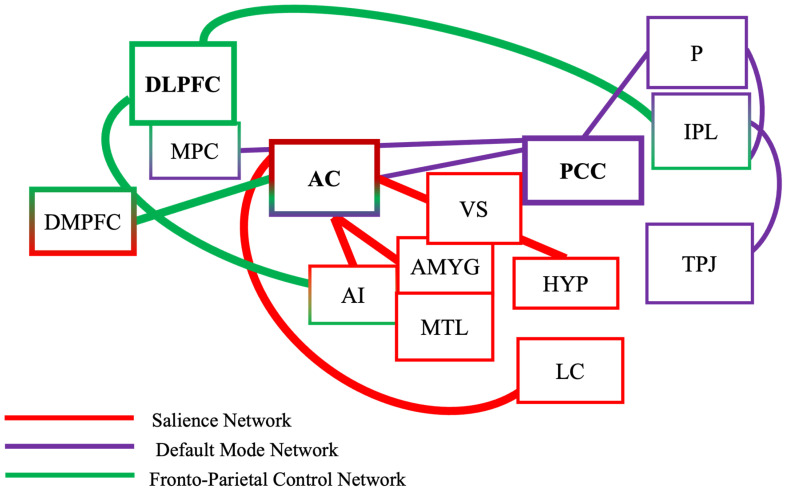
Graphic of anatomical connectivity of the DMN, SN, and the FPCN in dementia for working memory after music therapy. After music therapy, the activity in the DMN decreases (thinner line), and the activity in the FPCN and the SN increases (thicker line). DMPFC: dorso-medial pre-frontal cortex, DLPFC: dorsal–lateral pre-frontal cortex, MPC: middle pre-frontal cortex, AC: anterior cingulate, AI: anterior insular, MTL: medial temporal lobe, LC: locus coeruleus, AMYG: amygdala, VS: ventral striatum, HYP: hypothalamus, PCC: posterior cingulate cortex, P: precuneus, IPL: inferior parietal lobule, TPJ: temporal parietal junction. Nodes and hubs belonging to neural networks are designated by a coloured border, and hubs are designated by bold text. Nodes and hubs connected to more than one network have more than one colour in their border. Model based on previous work [36,37,66,67,68,69]. Figure adapted from [70].

This study aimed to improve the MMSE after music therapy for participants with dementia [54,64,65] and build on this research by completing an n-back task and obtaining novel neuroimaging data in the music therapy field by using magnetoencephalography (MEG) to observe whether cognitive benefits are associated with functional changes in the neural areas thought to underpin them. This study examined two hypotheses: (1) a music therapy (MT) program was designed to include working memory and executive tasks will improve working memory and executive functioning for participants with mild dementia, assessed by the n-back task, and (2) MT leads to measurable changes in neural activation and compensatory network recruitment in the FPCN and the SN in mild dementia. Figure 1 is a graphical representation of this hypothesis.

## 2. Materials and Methods

Participants with dementia were recruited through residential care located in Victoria Australia. All participants received a diagnosis of dementia from clinical and medical staff upon admission. Participants were excluded if their first language was not English, they had a history of claustrophobia, significant health comorbidities, hearing loss, visual impairments, or metal implants that posed risks to participants during the MRI scan, were wheelchair bound or diagnosed with psychosis, depression, or anxiety, or scored below 10 on the Standardised Mini Mental State Examination (SMMSE [71]). Participants were required to have no prior formal music training to control for prior neural plasticity and compensatory changes to the brain that may impact the interpretation of the MEG data. This study was conducted according to the guidelines of the National Statement on Ethical Conduct in Human Research and approved by Swinburne University Human Research Ethics Committee.

Ten participants were initially recruited. Two voluntarily withdrew (one after three weeks, the other after four weeks—these participants felt they were not contributing the therapy programs or the group and did not want to participate further), and two participants were excluded in week four on the advice of clinical care staff due to progressive cognitive decline during the study. The six remaining participants with dementia completed the MT: 2 males (*M* = 83.5, *SD* = 7.77) and 4 females (*M =* 86.3, *SD* = 6.62). Of these six participants, two were not eligible to participate in the neuroimaging component of the study due to medical confounds with neuroimaging scanners. Four participants completed all components of the study: MT, MEG and MRI scans, and the n-back task.

An age and gender-matched control group was recruited to measure against any change in the dementia group after the MT. This control group was recruited from a database of healthy older individuals who had previously participated in other research projects. Control participants were contacted by email and invited to participate. Interested individuals were emailed the Participant Information and Consent Forms. Three healthy older-age and gender-matched participants consented to participate in this study (the beginning of the COVID-19 pandemic and funding restricted completely matching dementia and control groups; see Table 1).

Informed consent for participants with dementia was initially obtained from their family member or guardian. If the participant was cognitively competent, as assessed by clinical staff at the residential home, consent was also obtained from participants. This consent process was completed by the residential home, as they had clinical knowledge of residents with dementia. Ongoing consent was also obtained verbally from the participants each time the researcher met with participants for clinical interviews, MEG scans, and each music therapy session.

### 2.1. Behavioural Measures

The SMMSE was used to screen participants, and they were excluded if scores were below 10, indicating severe cognitive deficits [71]. The dementia group completed the SMMSE before and after MT to measure any cognitive improvement after MT. The control group only completed the SMMSE before the MEG scans.

### 2.2. Music Therapy Procedure

The MT was developed and conducted by a registered music therapist with experience in dementia care settings. The face-to-face MT was run for one hour, one day a week for eight weeks, including a group performance on the eighth week. Each music therapy session was run in the afternoon, at the same time and in the same room, away from distractions. The design of the MT program was tailored for participants by developing rapport, mastery, and meaningful relationships between participants and the therapist as a way of improving therapeutic engagement and outcomes [72,73]. One staff member attended and participated in each music therapy session to provide clinical support for participants.

The MT was manualised, using activities and topics to engage participants across the eight weeks. Each music therapy session started with a song chosen by each participant. Participants then completed one of two working memory and executive functioning activities that focused on manipulating information using lyrics or drum rhythms. For the lyric activity, the therapist selected a familiar song. Before singing this song, the therapist asked participants to refrain from singing words starting with a certain letter (e.g., do not sing words beginning with the letter C). The song was sung by the therapist and participants once without the condition to establish familiarity with the rule, and then twice more omitting words beginning with the restricted letter. To increase the cognitive load, the therapist introduced another letter (e.g., do not sing words beginning with the letters C and M). For the rhythm activity, each participant received a drum and small mallet and was instructed by the therapist to hit the drum to 4/4 timing (four consecutive hits of the drum while counting one, two, three, four). To start, the therapist counted aloud 4 beats, hitting the drum at the same time and encouraging participants to play along. Once participants hit the drum consistently in time, the therapist asked participants to play every second beat (counts one and three). To increase the cognitive load, the therapist separated participants into two groups, one group played beats to counts of one and three (led by the music therapist), while the other group played beats to counts of two and four (lead by a researcher and guided by the therapist). These tasks required participants to hold information in memory, make decisions about information, and inhibit responses. The lyric and drum activities both ran for approximately 35 min and were completed in sessions two, three, four, five, six, and seven. In preparation for the performance in week eight, participants also learned the melody of “Doggy in the Window” [74] on tuning bells. For this activity, participants received the same tuning bell each time this activity was practiced so participants could master and take ownership of their note. To introduce this task, the therapist prompted each participant, starting from the first note of the song, when to play their bell, slowly creating the melody. This exercise required participants to monitor information, hold the previous note in memory, inhibit ringing the tuning bell until it was their turn, and ringing the tuning bell in a time. This task was practiced until each note was played with fluency and without guidance from the therapist. At the end of each therapy session, the therapist played a leaving song, marking the end of the session. In week eight, participants sung three songs (practiced in previous weeks) and performed “Doggy in the Window” [74] on tuning bells without guidance to their families, residential staff, and other residents. After the performance, participants were given certificates of completion signifying the end of the MT. The full protocol of the MT is described in the Supplementary Material.

### 2.3. N-Back Task

Participants with dementia completed the n-back during the MEG scan before and after MT, while the control group completed the n-back during the MEG scan once. Participants with dementia were escorted between the residential home and the university by two clinical staff members. The residential home was financially compensated for travel expenses.

**Figure 2:**
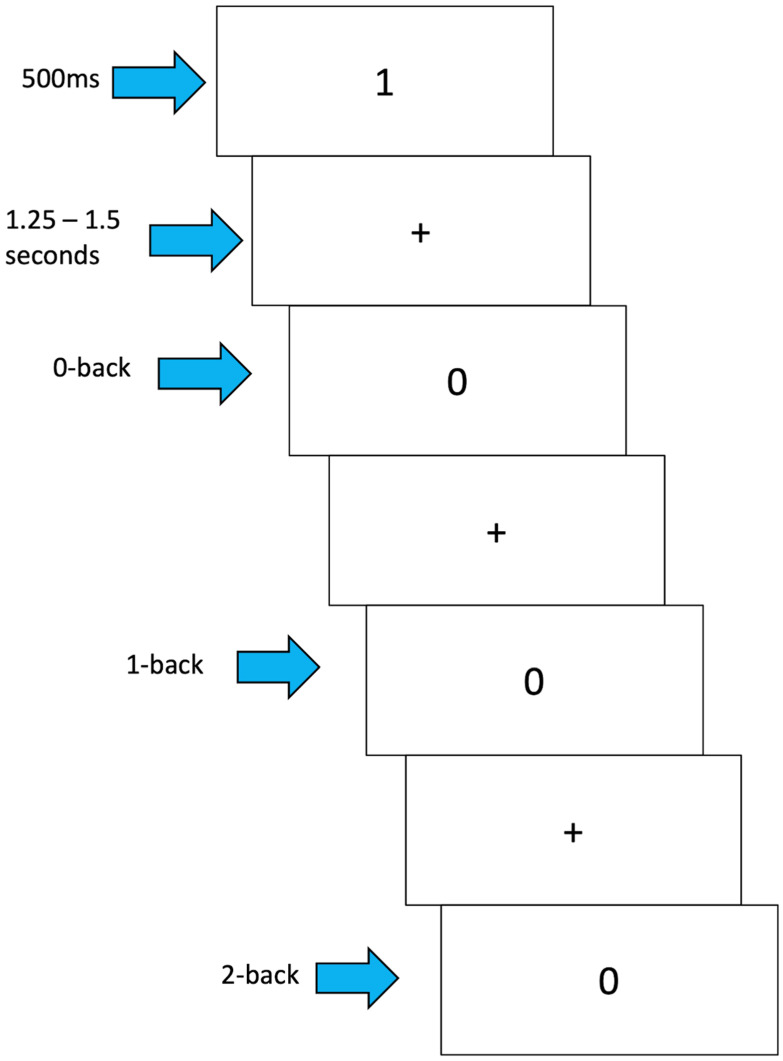
The N-back task.

The parameters of the n-back task were adapted from Fraga and colleagues [45]. To mitigate floor effects and fatigue or distress to participants, the n-back task was divided into two blocks of 300 stimuli (100 stimuli for each condition). The stimuli were white numerals ranging from 0 to 9, presented on a black background. There were three conditions: 0-back (control condition), 1-back, and 2-back. The n-back task was written and presented using PyschoPy (version 3.7 [75]). Each condition contained 100 trials, with 40 targets (0 s or matched numbers) and 60 non-targets (non-matched). Each number was presented for 0.5 s, followed by an inter-stimulus interval (ISI; fixation cross) with a random jitter between 1.25 s and 1.5 s. Overall, the design of the n-back task was constructed in an attempt to reduce participant fatigue, agitation, or distress. The total length of the n-back task was restricted to 25 min, and participants indicated when they were ready to start the first and second blocks. Each condition was presented twice (once in each block), pseudo-randomly to account for any learned effects across blocks and testing sessions. Correct, incorrect, and missed responses were recorded for target stimuli. Figure 2 shows the timing and structure of the three n-back conditions used in this study.

### 2.4. Magnetoencephalography

MEG data was recorded with the Elekta Neuromag (MEGIN, Keilasatama, Finland): Truix system at Swinburne University of Technology—306 sensors, organized into 102 sensor trios—1 magnetometer and 2 gradiometers. MEG scans were completed in a magnetically shielded room, Internal Active Shielding was off during data acquisition, and EOG and ECG were recorded for artifact removal. Online sampling was set to 1000 Hz, with an online bandpass filter between 0.1 Hz and 330 Hz.

Before scanning, participants were screened for metal implants (e.g., pacemakers, some stents, drains, artificial limbs, or joints) and claustrophobia to ensure participant safety and to protect the MEG sensors. As dementia often entails loss of episodic memory, reports of previous medical operations were checked against participants’ medical records and confirmed with their next of kin or medical power of attorney before the scan. During the MEG and MRI scans, participants were clinically supported by nursing staff from the residential home.

MEG scans were completed first because MRI scans can magnetize the human body, introducing electromagnetic noise into the MEG data [76]. With the help of the researcher and neuroimaging staff, participants completed a safety checklist and removed metal objects (belts, underwire bras, jewelry, watches, glasses, false teeth, clothing with metal buttons, etc.) before the scan. A noise test was conducted to assess signal quality before data was collected. If the MEG signals were free from visual artifact, five HPI coils were attached on the left and right mastoid and across the forehead to continuously track head movements. The Polyphemus Fastrak^®^ (Polhemus Inc., Colchester, VT, USA) system was used to digitize anatomical landmarks (nasion and left and right preauricular points), five HPI coils, and the shape of the skull. Lastly, eye sockets and the nose were digitized as reference points for co-registering the MEG and MRI scans. To remove artifact from eye blinks and the heart, electrodes were attached on the supraorbital ridge and the infraorbital ridge of the right eye to measure electrooculogram (EOG) and on the inferior side of the right and left wrist to record electrocardiogram (ECG), and a ground electrode was placed on the medial side of the right elbow.

To ensure participants were safe, the researcher and clinical staff escorted the participant into the scanning room and into the chair inside the MEG scanner. Once comfortable, the n-back task was set up. Participants did not require corrective glasses to complete the task. Once participants were comfortable, the instructions of the n-back task were explained.

### 2.5. Structural T1 MRI Acquisition

A 10 min structural T1 MRI scan was acquired for each participant so individual MEG data sensor locations could be co-registered to the anatomical areas on the surface of the same brain. One T1 MRI scan was collected for each participant and was used to analyse MEG data before and after the MEG scan. T1 images were acquired using a 3T Siemens TIM TRIO magnetic resonance imaging system at Swinburne University of Technology. Before the MRI scan, a safety screen was completed for each participant. Participants were escorted to the scanner by the radiographer and researcher. Participants were asked to keep their head still to prevent head movement and image blur. T1 weighted images were acquired in the sagittal plane with a magnetisation prepared rapid gradient echo (MP-RAGE) pulse sequence with an inversion recovery (176 slices, slice thickness = 1.0 mm, voxel resolution = 1.0 mm^3^, repetition time (TR) = 1900 ms, echo time (TE) = 2.52 ms, TI = 900 ms, FOV = 256 mm × 256 mm, orientation = sagittal, acquisition time = 10 min approximately).

### 2.6. Magnetoencephalography Analysis

All MEG data was pre-processed, filtered, and analysed using MNE Python Software (version 0.24) [77]. Before pre-processing, raw MEG data was co-registered with the T1 weighted structural MRI images by manually aligning the coordinate frame of the MRI fiducial points [78]. After the coordinate frames were aligned, the Boundary Element Model was calculated to determine the source amplitudes and surfaced-based source spaces. The source space was determined by FreeSurfer (Version 7) from the T1 images. The Boundary Element Model and source space were then used to compute the source space used to construct the forward solution.

#### 2.6.1. Pre-Processing

MEG data was visually inspected, and noisy and dead channels were removed from the raw data. If needed, data was then MaxFiltered using the default Eleckta MaxFilter (version 2.2, Helsinki, Finland) settings [79]. For some participants, two scans were recorded in one session. In these cases, scans were concatenated so adequate analysis and comparisons of the data could be made using the default head movement correction algorithm used by Elekta. Head movement was matched using the coordinates of the HPI coils from the first scan, with a step size of 200 milliseconds. The coordinates of the first scan were saved and replaced the HPI coordinates of the second scan, ensuring both scans had the same head movement coordinates, allowing concatenation after epoching.

MEG data was bandpass filtered between 1 Hz and 50 Hz, as guided by previous neuroimaging and n-back research [80,81,82] and MNE Python documentation [78]. Band pass filtering was completed before epoching to avoid edging effects with continuous MEG data [83]. ECG and EOG activity were then averaged and removed with MNE Python using the *fastICA* algorithm [84]. The number of components was set to 95% [85].

Noisy epochs were identified by computing and comparing different standard deviations (e.g., three, four, five, and six) of the mean peak-to-peak amplitudes to identify a noise rejection threshold. Epochs above this threshold (noisy epochs when compared to the average noise in the data) were removed. As magnetometers and gradiometers have different recording sensitivities [86,87], the standard deviations of the peak-to-peak threshold were computed separately for the magnetometer and gradiometers for each participant and for each scan.

MEG data was then epoched from 500 milliseconds before and 1.5 s after the onset of the target stimuli [45,88]. Some participants held the response button after responding to target stimuli, creating an accumulative effect of event values. In these cases, the event value data was cleaned to only contain the trigger and single button press values. Epochs for correct and incorrect responses (unexpected button presses or missed responses after the target stimuli) were created from the event values. Correct epochs were used to analyse differences in functional neural network connectivity between MEG scans before and after music therapy. Guided by the data, and previous electroencephalography (EEG) and n-back research [45], epochs were cropped from 0 ms to 1300 ms after the trigger. From the correct epochs, a noise covariance matrix was produced to solve the inverse problem.

#### 2.6.2. Connectivity

The neural connectivity patterns before and after MT were compared for the dementia group. Source localisation of neural activity was determined by computing the inverse solution with the inverse operator. The inverse operator was computed using the covariance matrix and the forward solution. Default MNE Python settings were used to compute the inverse operator: the loose (value weight) was set to 0.2, with a depth weighting exponent of 0.8 [77]. The inverse operator was then applied to the source space by averaging the source estimates within anatomical labels using mean_flip to reduce any signal cancellation. Anatomical areas were parcellated into 68 labels using FreeSurfer. The inverse solution was computed using dynamic statistical parametric mapping [89] and was applied to each epoch. For each participant in the dementia group, the 300 strongest connections were calculated for theta, alpha, beta, and gamma frequency bands before and after MT during the n-back task. Connectivity was calculated using default settings in MNE Python [77], which required the source estimates to be averaged across all points of the 68 regions in the FreeSurfer segmented parcellations. The weighted phase lagged index squared debiased connectivity method was used to produce the connectivity circle plots [90]. The specific steps of the MEG analysis are presented in the Supplementary Materials.

#### 2.6.3. Statistics

Two-tailed *t*-tests determined if there were significantly stronger and weaker connectivity differences between the 300 strongest connections before and after MT. This statical analysis was completed for each participant, as group numbers were too small for averaging epochs across groups. For theta, alpha, beta, and gamma bands, the mean connectivity adjacency matrix across all epochs was calculated, and the standard error of the mean was estimated using a leave-one-out jack-knife procedure.

For each participant, the differences of the means and the pooled standard errors were used to calculate a *t*-statistic and *p*-value for each neural connection in the connectivity matrix, contrasting connectivity after music therapy with connectivity before music therapy. To control for multiple comparisons, *p*-values were corrected with the False Discovery Rate (FDR) procedure [91,92] with the threshold set to *q* < 0.05. A *q*-value statistic was calculated from significant neural connections to determine how many significant findings were due to Type I error [92]. A connectivity plot was produced using the significant connections that survived the calculated *q*-value statistic to show significantly stronger and weaker connectivity between scans taken before and after MT for each participant.

## 3. Results

### 3.1. SMMSE and N-Back Task

SMMSE scores decreased after music therapy, but this change was non-significant *t*(4) = 0.845, *p* = 0.45. A two-tailed *t*-test with a Bonferroni adjusted alpha of 0.025 revealed non-significant differences between the SMMSE scores before MT and the scores of the SMMSE for the control group (*t*(5) = 2.054, *p* = 0.95), and between the SMMSE scores after MT and the SMMSE scores for the control group (*t*(4) = 2.409, *p* = 0.74).

Table 2 shows the mean total correct, wrong, and missed responses for the n-back task for the 0-back, 1-back, and 2-back conditions before and after the music therapy program for the dementia group. The total mean number of correct n-back scores was higher after the music therapy program. The total mean number of wrong n-back responses was lower after the music therapy program, and the total mean number of missed n-back responses was also lower after the music therapy program.

Table 2 shows the averaged total correct, incorrect, and missed responses for the three n-back conditions for the dementia group before and after music therapy and the average total correct, incorrect, and missed responses for the n-back task across the three conditions for the control group. For the dementia group, Table 3 shows three paired *t*-tests with a Bonferroni corrected alpha of 0.016, assessing differences between correct, incorrect, and missed responses before and after music therapy. After music therapy, a non-significant increase in correct responses *t*(3) = −1.394, *p* = 0.26, a non-significant decrease in incorrect responses *t*(3) = 1.569, *p* = 0.22, and a non-significant decrease in missed responses *t*(3) = 0.280, *p* = 0.80 were shown.

**Figure 3:**
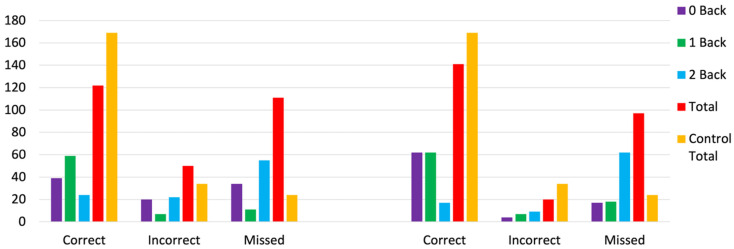
Average correct, incorrect, and missed N-back scores for the dementia before and after music therapy and control group.

Figure 3 shows the average correct, incorrect, and missed responses on the n-back task for the dementia before and after music therapy. Scores before and after music therapy are then compared with the age matched control group. As shown in Figure 3, correct scores generally increased and incorrect scores decreased (closer to the control group) after music therapy.

Shapiro Wilk tests of normality show the null hypothesis for normality was not rejected for the SMMSE before and after music therapy for the dementia group. The Wilcoxon Signed Rank Test shows the differences between the pre-rank tests and post-rank tests; the SMMSE (*Z* = 10, *p* = 0.59) was not significant and therefore did not reject the null hypothesis, indicating no evidence for a departure from normality. The null hypothesis for normality was not rejected when the SMMSE scores for the control group were compared before (*Z* = 0.964, *p* = 0.64) and after (*Z* = 0.998, *p* = 0.92) MT.

Shapiro Wilk Tests of normality provided no evidence for non-normality for the dementia group when comparing before and after MT for correct, incorrect, and missed responses. The Wilcoxon Signed Rank Test shows the post-MT overall correct (*Z* = 2, *p* = 0.38), overall incorrect (*Z* = 9, *p* = 0.25), and overall missed (*Z* = 6, *p* = 0.88) n-back scores before and after MT were not significant.

### 3.2. Comparisons of N-Back Scores Between Dementia and Control Groups

Table 3 shows nine paired *t*-tests with a Bonferroni adjusted alpha of 0.008, effect sizes, and confidence intervals (CI). For correct responses, the differences between the control group and the pre-music therapy scores *t*(2) = 1.409, *p* = 0.29 (d’ = −0.813, CI = −2.106, 0.596), and post-music therapy scores was non-significant *t*(2) *= −*0.537, *p* = 0.465 (d’ = −0.31, CI = −1.443,0.889). For incorrect responses, the differences between the control group and the pre-music therapy scores, *t*(2) = 0.652, *p* = 0.418 (d’ = 3.396, CI = 0.233, 6.683), and post-music therapy scores, *t*(2) = −1.519, *p* = 0.286 (d’ = −0.087, CI = −2.202, 0.566), were non-significant. For missed responses, the differences between the control group and pre-music therapy were significant *t*(2) = 5.882, *p* = 0.0028 (d’ = 0.361, CI = −0.854, 1.502), and between the control group and post-music therapy, scores were non-significant *t*(2) = 2.834, *p* = 0.105 (d’ = 1.63, CI = −0.260, 3.459).

The Shapiro Wilk tests of normality shows the null hypothesis for normality is not rejected for the correct, incorrect, and missed responses when comparing the control group and the dementia group before and after music therapy (Table 3). The Wilcoxon Signed Rank Test shows that the pre-rank tests and post-rank tests for the correct (*Z* = 2, *p* = 0.38), incorrect (*Z* = 9, *p* = 0.25), and missed responses (*Z* = 6, *p* = 0.88) were not significant (Table 3). This test was used as there were unequal groups sizes when comparing the dementia and control groups.

### 3.3. Standard Detection Theory Sensitivity Measure d’

Response rates and percentage correct responses varied greatly across participants, conditions, and trial type. Much of this variability is likely due to age-related effects and disease. The percentage of correct and incorrect responses is shown in Table 4. Despite adjustments to the task, in many cases, participants responded slowly and tended to miss trials. This makes it hard to judge from raw response counts whether participants are responding above chance or not. A statistic which adjusts for response rate (especially very high or very low rate) while also considering errors (e.g., many correctly identified targets means little in the context of many errors) is the standard signal detection theory (SDT) sensitivity measure d’ [93]. This is calculated as the proportion of hits (correct identifications of an n-back target) converted to a Z-score minus the proportion of false alarms (incorrectly responding on a distractor trial) converted to a Z-score. A d’ > 0 indicates that the participant is making more correct identifications than errors. For most detection tasks with healthy participants, a d’ > 1.35 is often considered “good” in reflecting many correct identifications and relative errors. d’ for participants is shown in Table 4.

Dementia patients showed the lowest overall performance in the 2-back condition, indicating it was the hardest task. Negative d’, indicating making more errors than correct responses, was observed for Participant 3 pre and Participant 4 post-intervention. Interestingly, they performed quite better in 1-back than the 0-back condition. There appeared to be a marginal improvement in 1-back post-intervention and marked improvement in 0-back post intervention. The control performed consistently across all conditions, and performance was generally better than the patient group (about the same in 1-back).

### 3.4. Reliable Change Statistic: SMMSE and the N-Back Task

As the dementia group was small, the Reliable Change Index (RCI) statistical test was conducted to assess any significant differences between individual scores after music therapy for the SMMSE and the total correct, incorrect, and missed response on the n-back task. The RCI determines whether the magnitude and direction of change of an individual’s score are statistically reliable [94]. The following Equations (1)–(3) for determining the RCI were developed by Jacobson et al. [95] and refined by Christensen and Mendoza [96]. The RCI was determined by calculating the standard error ($S_{E}$) and the spread of data distribution of change scores $\left(S_{diff}\right)$.

$S_{E}$ is the standard error and is calculated by Equation (1). $S_{1}$ is the standard deviation of the scores of the dementia participants (MMSE and the n-back task) after music therapy, *r_xx_* is the test–retest reliability score.

(1) $$S_{E}=S_{1}\sqrt{1-rxx}$$

The $S_{diff}$ is the spread of data of distribution of change scores if no change had occurred and is calculated by Equation (2).

(2) $$S_{diff}=\sqrt{2{\left(S_{E}\right)}^{2}}$$

The RCI statistic is determined by Equation (3). *X*1 is the raw score before music therapy, and *X*2 is the raw score after music therapy.

(3) $$\mathrm{RCI}=\frac{X2-X1}{\sqrt{S_{diff}}}$$

If the RCI exceeds +1.96 or −1.96, there is a 5% chance the change in scores is unreliable—the RCI is reliable to the level of *p* = 0.05 [94]. Test–retest reliabilities were used in Equation (1): 0.92 for the SMMSE [71], and 0.82 for the number n-back task [97].

The RCI statistics for the SMMSE for individual dementia participants before and after music are shown in Table 5. For the SMMSE, scores significantly decreased for two participants, significantly increased for two participants, and non-significantly increased for a fifth. The SMMSE score after music therapy was missing for the final participant, and the RCI could not be calculated.

The RCI for the total correct, incorrect, and missed n-back responses for individual dementia participants before and after music therapy across the three conditions is shown in Table 5. Significant changes were shown for total correct, incorrect, and missed responses after MT. For correct responses, two participants significantly improved, one participant showed a non-significant increase, and one participant showed a non-significant decrease after MT. For incorrect responses, scores significantly decreased for three participants, while there was no difference for one participant. For missed responses, scores significantly decreased for two participants, significantly increased for one participant, and non-significantly decreased for one participant.

Significant changes were also observed across the three conditions for the total correct, incorrect, and missed responses after MT. For correct responses, in the 0-back condition, two participants significantly improved, non-significantly increased for one participant, and non-significantly decreased for one participant. For the 1-back condition, scores significantly increased for one participant, non-significantly increased for one participant, non-significantly decreased for one participant, and one participant did not show a change. For the 2-back condition, scores significantly increased for one participant, non-significantly increased for one participant, and significantly decreased for two participants.

For incorrect responses, in the 0-back condition, scores non-significantly increased for one participant, significantly decreased for one participant, and non-significantly decreased for two participants. For the 1-back condition, scores non-significantly decreased for two participants and non-significantly increased for two participants. For the 2-back condition, scores significantly decreased for one participant and non-significantly decreased for three participants.

For missed responses, in the 0-back condition, scores significantly decreased for three participants and non-significantly increased for one participant. For the 1-back condition, scores significantly decreased for one participant, significantly increased for one participant, non-significantly increased for one participant, and one participant showed no change. For the 2-back condition, scores significantly increased for two participants and non-significantly decreased for two participants.

### 3.5. Magnetoencephalography

**Figure 4:**
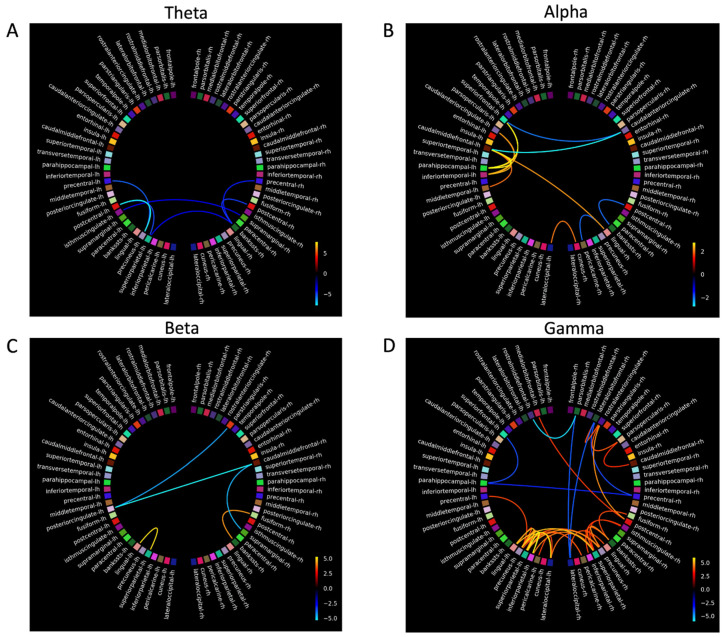
Significant connectivity differences between before and after music therapy for participant 2 for theta, alpha, beta, and gamma bands. (**A**) Significant connectivity differences show FDR corrected *q*-values in the theta band, (**B**) significant connectivity differences of uncorrected *p*-value scores at <0.05 in the alpha band, (**C**) significant connectivity differences show FDR corrected *q*-values in the beta band met the FDR correction, (**D**) significant connectivity differences show FDR corrected *q*-values in the Gamma band.

**Figure 5:**
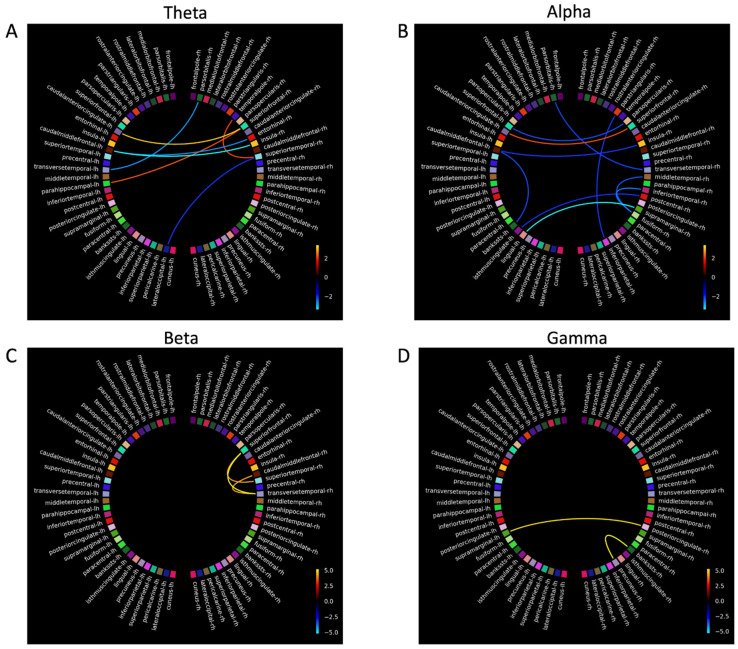
Significant connectivity differences between before and after music therapy for participant 3 for theta, alpha, beta, and gamma bands. (**A**) Significant connectivity differences of uncorrected *p*-value scores at <0.05 in the theta band, (**B**) significant connectivity differences of uncorrected *p*-value scores at <0.05 in the alpha band, (**C**) significant connectivity differences show FDR corrected *q*-values in the beta band, (**D**) significant connectivity differences show FDR corrected *q*-values in the gamma band.

**Figure 6:**
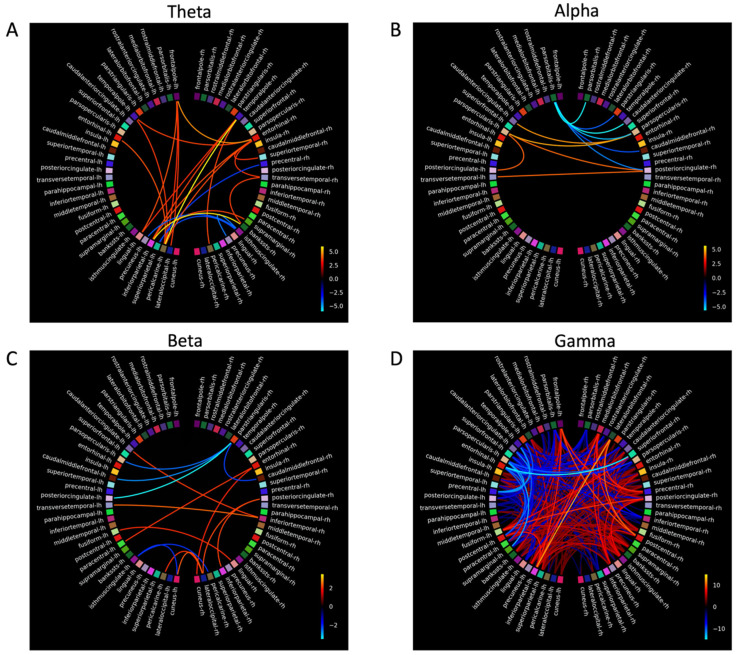
Significant connectivity differences between before and after music therapy for participant 4 for theta, alpha, beta, and gamma bands. (**A**) Significant connectivity differences show FDR corrected *q*-values in the theta band, (**B**) significant connectivity differences show FDR corrected *q*-values in the alpha band, (**C**) significant connectivity differences of uncorrected *p*-value scores at <0.05 in the beta band, (**D**) significant connectivity differences show FDR corrected *q*-values in the gamma band.

**Figure 7:**
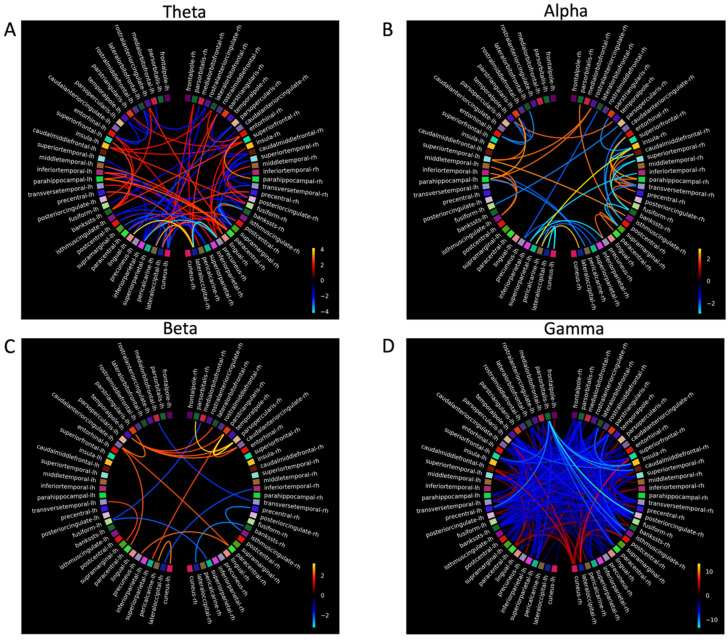
Significant connectivity differences between before and after music therapy for participant 5 for theta, alpha, beta, and gamma bands. (**A**) Significant connectivity differences of uncorrected *p*-value scores at <0.05 in the theta band, (**B**) significant connectivity differences of uncorrected *p*-value scores at <0.05 in the alpha band, (**C**) significant connectivity differences of uncorrected *p*-value scores at <0.05 in the beta band, (**D**) significant connectivity differences show FDR corrected *q*-values in the gamma band.

Four participants with dementia and three control participants completed MEG scanning. The dementia group completed MEG scans before and after MT. Due to the small sample size in the dementia and control groups, MEG analyses were treated as case studies. The connectivity plots in Figure 4, Figure 5, Figure 6 and Figure 7 show *t-scores* of connectivity that were significantly different between the MEG data acquired before and after MT for each participant in the dementia group across theta, alpha, beta, and gamma frequency bands. When the FDR correction was applied to all *p*-values, some connectivity analyses did not meet the FDR correction threshold of *q* < 0.05 and were therefore plotted using the significant uncorrected *p*-value < 0.05 (Table 6).

The coloured lines shown in the connectivity plots (Figure 4, Figure 5, Figure 6 and Figure 7) represent the significance of the two-tailed *t*-test at *p* < 0.05: orange colours in the plot spectrum represent significantly stronger connectivity, while more blue colours represent significantly weaker connectivity for the four frequency bands. The larger scale on Figure 5 and Figure 6 reflects a greater significant difference at *p* < 0.05 in *t-scores* between the MEG scans before and after MT in the gamma band.

#### 3.5.1. Theta

Connectivity differences in the theta band were significantly stronger between the anterior SN (rostral anterior cingulate (RAC), entorhinal cortex (EC), superior frontal gyrus (SFG), and the insular (IN)) and the DMN and between the frontal (frontal pole (FP)) and occipital lobes (pericalcarine (PCL), cuneus (C), the pars orbtitalis (POB), and the lateral occipital sulcus (LOS)). Significantly weaker connectivity was shown between the posterior SN (superior temporal gyrus (STG), inferior temporal gyrus (ITG) and the precentral gyrus (PG)) and the DMN (the banks of the superior temporal sulcus (BSSTS), the lingual gyrus (LG), precuneus (P), and the superior parietal lobule (SPL)). Significantly weaker connectivity differences were shown within the FPCN (between the temporal pole (TP) and the medial orbital frontal (MOF), between the TP and the lateral orbital frontal gyrus (LOF), between the TP and the LOF, and also within the inferior frontal gyrus: POB, pars triangularis (PTL), and the pars opercularis (POL)) within the DMN (between the fusiform gyrus (FG) and the P, between the post central gyrus (PCG) and the BSSTS, between the isthmus cingualte [IC] and LG, between the IC and inferior parietal lobule (IPL), and between the PCG and the IPL), and between the C, PCL, and the LOS.

#### 3.5.2. Alpha

For the alpha band, significantly stronger connectivity differences were observed within the SN (caudal anterior cingulate (CAC), EC, IN, caudal middle frontal gyrus (CMF), PG, STG, and the transverse temporal gyrus (TT)). Significantly weaker connectivity differences were seen within the FPCN between areas of the IFG (PO, PTL and POL), between the FPCN (SFG) and the SN (CAC), and within the DMN (between the FG and the LG, between the PCG and the LG, and between the LG and P). Other areas of significant connectivity not directly associated with the FPCN, SN, or DMN included the C, PCL, and the LOS.

#### 3.5.3. Beta

In the beta band, significant connectivity differences, generally bilateral and fewer compared to other frequency bands, were shown in the frontal, temporal, and parietal lobes. Significantly stronger connectivity differences were shown within the FPCN (between the TP and LOF, between the TP and PTL, between the TP and POL, and between the TP and the SN (CAC and the EC)). Significantly stronger connectivity differences were shown within the DMN (between the FG and the LG), and within the SN (between the PC, IPL, paracentral gyrus (PACG), and the TT). Significantly weaker connectivity was shown between FPCN (PTL) and the SN (CAC, CMF), and the FPCN (PTL) and the DMN (PC), and within the DMN (between the PACG and the IPL and between FG and the P).

#### 3.5.4. Gamma

Significant connectivity differences in the gamma band were shown within the DMN and between the FPCN and the DMN. Significantly stronger connectivity differences were shown within the DMN (between the PC and FG, between the PC and IC, and between the PC and the supramarginal gyrus (SMG) between areas of the FPCN (FP, LOF) and the SN (PCG), and between areas of the DMN (IPL). Significantly stronger connectivity differences were also shown between the DMN (SPM, PACG, BSSTS, and the P) and the C and LOS. Significantly weaker connectivity differences were shown bilaterally between the IFG (POB) and the SN (IN, CMF) and between the IFG and the DMN (PC, PCG, IMC, and the PCG). Significantly weaker ipsilateral connectivity differences were observed between the POB and the LOS, between the LOF and LOS, between the SFG and the LOF, between the PRCG and the POL, and between the IMC and the PCG.

Participants 4 and 5 had large significant connectivity differences in the gamma band compared to other participants. Due to the large number of significant results, there was a chance the Storey [98] FDR correction may have rejected more null hypotheses [99]. To test this, the connectivity plots for participants 4 and 5 in the gamma band were calculated using the FDR correction by Benjamini and Hochberg [91] and the uncorrected *p*-values at <0.05. The connectivity plots using the Benjamini and Hochberg [91] FDR correction and the uncorrected *p*-values were similar to the Storey [98] FDR correction, suggesting the large significant differences in the gamma band for participants 4 and 5 were meaningful.

## 4. Discussion

This study intended to improve the SMMSE scores for participants with dementia and obtain MEG data to measure any changes to functional activity in the brain after MT. Two specific hypotheses were assessed: (1) a working memory and executive functioning structured MT will improve inhibitory control, working memory, and executive functioning for a dementia group, assessed by the n-back task, and (2) this music therapy intervention leads to measurable changes in neural activation and compensatory network recruitment in the FPCN and the SN.

This study did not improve the SMMSE for participants with dementia, suggesting global cognition did not change after the MT program The first hypothesis was supported in part, as participants with dementia generally showed some improvement on the n-back task after music therapy. The second hypothesis showed stronger support, as significantly stronger connectivity was shown in areas of the FPCN and the SN, suggesting, with caution due to the small sample sizes, that compensatory activation may occur after MT for people with dementia.

### 4.1. RCI Statistic for the SMMSE

The individual SMMSE scores varied but generally did not improve after MT for participants with dementia. This finding is contrary to previous music therapy research with dementia patients [54,65,100,101]. Although the MMSE shows good short-term re-test reliability of 1 month, due to age-related cognitive decline, measurement errors at the first time point of data collection, and increasing clinical heterogeneity of participants [102,103], re-test reliability decreases with time due to disease progression [104]. Furthermore, the variability of individual MMSE scores is associated with the number of comorbidities and their symptoms, suggesting one’s ability to complete components of the MMSE may not necessarily due to cognitive decline but would result in a varied or lower score (e.g., hand tremors would impact one’s ability to copy the overlapping hexagons task or hearing impairments would hinder the interpretation of questions [103].

Short-term variation of individual MMSE scores is also evident in Alzheimer’s disease, as 22% of individual MMSE scores can differ by more than 3 points two weeks after baseline due to measurement errors and cognitive fluctuations [105]. As the MT program intended to improve cognitive functioning, individual variations on the SMMSE may not reflect changes in cognitive functioning but are measuring cognitive fluctuations. Cognitive fluctuations (e.g., behavioural confusion, inattention, and variable performance on cognitive tasks) are common in people with dementia and impact psychometric testing, accounting for between 3% and 11% of the variation in global cognitive performance [106].

### 4.2. N-Back Task

Working memory performance and executive functioning, as assessed by the n-back task, improved somewhat after MT in the dementia group, although this improvement was not significant. This trend in our results is consistent with previous studies of learning and transfer effects in people with dementia after a training program [14,15,51,52,53]. Although the differences between the n-back scores before and after MT were not significant for dementia participants, this trend (more correct, and fewer incorrect and fewer missed responses), suggests MT used in this study may improve symptoms of working memory, executive functioning, attention, and inhibitory control in dementia

The results of the SMMSE and the trends of the n-back task scores after MT for the dementia group suggest MT specifically designed to target cognitive impairments in dementia (e.g., working memory) may be more effective in improving specific cognitive functions rather than global cognition (e.g., SMMSE). Previous empirical research using music therapy to improve symptoms of dementia supports this [7,64,107,108], suggesting MT for people with dementia can be developed to intentionally target and improve specific cognitive processes.

### 4.3. RCI Statistic for the N-Back Task

The total individual scores after MT saw two participants in the dementia group make reliably more correct responses, three participants made reliably fewer incorrect responses, and two participants reliably missed fewer targets. Individual scores indicated that the 2-back condition was more challenging for participants with dementia. In the 2-back condition, two participants had a reliable decrease in correct responses, and two participants had a reliable increase in missed responses after MT. The number of missed responses in the 2-back condition after music therapy in the dementia group accounted for one-third of all possible target stimuli compared to the 0-back and 1-back conditions. The increased incorrect and missed responses in the 2-back for the dementia group may reveal differences in attention with cognitive load. As the 2-back condition requires a longer attention span (holding more information in working memory and attending to more stimuli), participants with dementia may have had difficulty sustaining their attention between two targeted stimuli in the 2-back condition. Empirical evidence suggests people with AD make more mistakes when responding to continuous tasks with increased cognitive load compared to tasks without an increasing cognitive load [23]. Lapses in sustained attention are associated with decreased working memory and executive functioning performance [109], are associated with dysfunctional connectivity of the DMN during trial-by-trial cognitive tasks [27], and are associated with neuropathology within the LC [110].

The control group outperformed the dementia group on the n-back task by identifying more correct targets, making fewer incorrect responses, and missing fewer targets. These findings replicate research examining n-back results between healthy older and dementia groups [46,47,111]. The improvements in correct responses and the decreases in incorrect and missed responses after music therapy for the dementia group are promising findings, as they suggest working memory and executive functioning performance can be improved towards a cognitive level of age and gender-matched healthy older persons. Based on the association between completing the n-back task and FPCN activity [112], it could be inferenced that the improved performance (RCI statistic) on the n-back task for some participants in the dementia group suggests MT increased the activation or improved the efficacy of neural networks. For example, the n-back task activates the FPCN [29,42], a network involved in working memory, executive functioning, and attention [69], shows compensatory activation to maintain cognitive functioning in AD and dementia Pariente [20,113], and is likely to be activated by music therapy directly or by other pathways such as the SN [70].

Although the progression of dementia will likely outrun cognitive improvements via compensation [40,114], greater improvements in working memory and executive functioning in dementia may be observed with longer and more intensive MT.

### 4.4. Neural Connectivity

The hypothesis that music therapy can increase the activation of the compensatory activity of the FPCN and the SN was partly supported. There is some evidence supporting these findings [50], but to our knowledge, this is the first study that has assessed functional neural connectivity differences in dementia participants in response to a cognitive intense MT program measured with MEG.

### 4.5. Connectivity Differences Before and After Music Therapy

For participants with dementia, analysis comparing connectivity between the MEG scans before and after MT revealed significant functional connectivity changes in areas that are associated with working memory and executive functioning [21]. Overall, stronger connectivity was shown in temporal and posterior areas of the SN and DMN, and significantly weaker connectivity was shown in the frontal areas of the FPCN. These connectivity differences between MEG scans taken before and after MT show complex interactions between neural networks and frequency bands, suggesting targeted music therapy can change functional neural connectivity in dementia.

### 4.6. Connectivity Differences in Theta, Alpha, Beta, and Gamma Frequency Bands

To further examine the connectivity results of this study, each frequency band will be addressed separately, as significant connectivity differences did not mean the FDR thresholds varied in the hubs and nodes of the DMN, FPCN, and the SN for each frequency band. In addition, for each frequency, band stronger and weaker connectivity was shown in neural areas not immediately associated with the FPCN, SN, and the DMN and may have a functional influence on their activation patterns. For clarity, only results that met the FDR threshold will be reported on.

#### 4.6.1. Theta

Theta power increases in response to increasing working memory loads, particularly in the right hippocampus [115]. It is modulated when controlling working memory, suggesting theta may integrate functions of distributed neural networks (e.g., FPCN) [116].

Theta band connectivity was significantly weaker (at the FDR threshold) for two participants in areas of the FPCN, DMN, in occipital regions, and between the P and the IMC and IPL. Decreased connectivity in the theta band in areas of the FPCN, DMN, and occipital lobes have been observed to be associated with healthy older adults performing working memory tasks [117].

Significantly stronger connectivity (at the FDR threshold) in the theta band was shown within the SN and between the SN and DMN. EEG evidence suggests neurofeedback training that increased front-to-midline theta in older adults improves performance on the working memory and attentional tasks [118]. Fronto-midline theta is generated in the anterior cingulate (the hub of the SN [119]) and the superior frontal gyrus [80], suggesting increased connectivity in the theta band within the SN is a compensatory process [34] and, when enhanced by MT, could contribute to improving performance on a n-back task. Specifically, the significantly stronger connectivity between the EC and the SPL and between the EC and IPL may reflect inter-network interactions [120,121]. This connectivity pattern in the theta band is observed during video game training with healthy older adults, which saw increased theta power between the middle temporal lobes (MTL) and the posterior lobes, leading to improvements in working memory and attention [122]. This suggests targeted cognitive training can improve working memory and executive functioning by increasing activation between middle temporal and parietal areas.

After music therapy, significantly stronger connectivity was observed in the FP, an area functionally connected to the DLPFC [123], and in the TP, an area associated with memory processing and activation during the n-back task [124,125] and is positively correlated with n-back performance [126]. The PCL and LG also showed significantly stronger connectivity and may reflect visual processing in response to the task [127,128]. The PCL is the primary visual area of the occipital cortex, and in response to visual working memory processes, it then connects with other areas of working memory processes such as those in the TP and the EC [129].

#### 4.6.2. Alpha

Alpha power increases with increased task loads [130], reflecting inhibition of neural areas unrelated to task performance [131]. In AD, resting state peak alpha power is lower compared to aged-matched controls [132] and increases in frontal, parietal, and occipital regions when completing the n-back task [45].

In our study, significantly stronger connectivity differences (at the FDR threshold) in the alpha band are shown within the SN and between the SN and DMN, and weaker between the FPCN and SN. As alpha is generated from the anterior cingulate [133], an area known to process music in dementia [61]), alpha power may be a therapeutic target to improve and maintain cognitive performance in dementia.

The connectivity findings that met the FDR threshold show activation of the CMF, the anterior and posterior cingulate, the POL, and POB. Activation of the CMF is associated with reorientating attention to novel stimuli (such as the target stimuli of the n-back task) and is functionally connected to the FPCN [134]. While activation of the POB is a node associated with the FPCN and the DMN [135], the POL is the caudal part of the inferior frontal gyrus and activates when identifying targeted stimuli [136] and inhibiting response to stimuli [137]. The connectivity patterns seen in the CMF, the POB, and the POL may indicate these areas are recruited by neural networks or are activated by music therapy to further support working memory and executive functioning performance in dementia.

#### 4.6.3. Beta

In dementia, beta band activity decreases during working memory tasks [138]. This pattern of activity in the beta band was shown in this study, as there were fewer significant connectivity differences and less intra- and inter-hemispheric connections observed to the other frequency bands during the n-back task.

Specifically, the significantly stronger connectivity (at the FDR threshold) in this study is supported. Activity in the superior frontal gyrus is shown to increase connectivity after working memory training [139], and the traverse temporal gyrus shows hyper activity in dementia when completing complex working memory tasks [140]. Activity in the superior temporal gyrus and the superior frontal gyrus also increases in response to cognitive training, participants in the initial response to cognitive training in the beta band were shown within the DMN and SN.

Activation in the MTL in our study was evident but should be interpreted with caution, as some of these connections (participants 4 and 5) did not meet the FDR threshold. These results were unexpected given the hippocampus, PG, and the EC (areas located within the MTL) are the first areas to show neural atrophy in AD [141,142,143]. However, neuroimaging evidence with MCI participants suggests MTL activation during working memory tasks is a compensatory response that maintains cognitive functioning [144,145]. Furthermore, as beta activity in the MTL facilitates holding and retrieval of visual working memory stimuli [146], and activation of the EC and PG are associated with familiarity of stimuli and information retrieval, respectively [147], the connectivity of the MTL after MT may have been compensatory, facilitating the recognition and retrieval of n-back stimuli, and could contribute to the improved performance on the n-back task after MT for the dementia group, although this needs further investigation.

The significantly weaker connectivity in the IFG found in this study was not replicated in the previous dementia literature, as findings suggest increased IFG activation is a compensatory response to AD pathology [148] and decreased activation of LC and norepinephrine systems [149]. The significantly weaker connectivity within the IFG, and between frontal areas and the posterior DMN may reflect variability in frontal activation in response to working memory tasks. A study by Kochan and colleagues [150], with MCI participants, suggests the activation of the CAC and the P (the hub of the DMN), increases with low cognitive loads and decreases with high cognitive load. Although connectivity analysis was not conducted for each n-back condition, the weaker connectivity shown within the FPCN, and between the FPCN and the posterior DMN, may reflect a compensatory response to the increased cognitive load of the 2-back by allowing greater activation in the SN, preserving cognitive resources, and managing increased cognitive loads—this compensatory hypothesis needs further testing [150,151].

#### 4.6.4. Gamma

Gamma power increases with working memory load [152] and is positively correlated with the number of target stimuli held in working memory [153]. Gamma power increases at rest over the parietal and occipital areas of the brain in MCI [154] and AD, suggesting a compensatory response to dysfunctional activity of other frequency bands in other neural areas [155].

In our study, significant connectivity differences at the FDR threshold within the gamma band show large variations between participants. Increased interhemispheric gamma power in parietal and occipital regions during a verbal working memory task in MCI cohorts is suggested to be compensatory for dysfunctional activity in other areas of the brain [154]. For other participants in this study (4 and 5), there was widespread significant connectivity changes between neural areas. Connectivity in the gamma band shows high heterogeneity between participants with AD [156], where connectivity between fronto-parietal areas is usually long-range compared to other bands during attention tasks [157].

The widespread high coherence of connectivity in the gamma band for participants 4 and 5 may be to reduce hyper-synchronization of neural activity, improving memory encoding and retrieval [158]. Furthermore, the widespread connectivity in gamma may be related to dysfunctional inhibitory interneurons, resulting in decreased activation of GABAergic neurons and less cortical inhibition [158] and may reflect as a compensatory response to other dysfunctional neural networks in dementia [157]. Gamma power correlates with cognitive performance and may indicate functional neural plasticity and improvements to cognitive functioning for people with MCI and AD [159,160] in response to a physical exercise and cognitive training [161]. This evidence suggests the results in the gamma band found in this study are meaningful, may support neural compensation in response to cognitive tasks, and may also support cognitive improvements on tasks like the n-back in response to training.

### 4.7. Proposed Model of How Music Therapy Changes Connectivity

Figure 4, Figure 5, Figure 6 and Figure 7 suggest an interplay of stronger and weaker oscillatory activity across three large-scale networks during the n-back task after MT in a dementia group. The significant connectivity differences in the FPCN, IPL, P, CAC, and IN in this study are also consistent with neural areas activated by the n-back task in healthy older adults [162]. In addition, the significantly stronger connectivity in SN, particularly in the areas of the MTL, IN, and CAC, suggests the increased activation of the SN had an overall compensatory effect [162] after music therapy.

**Figure 8:**
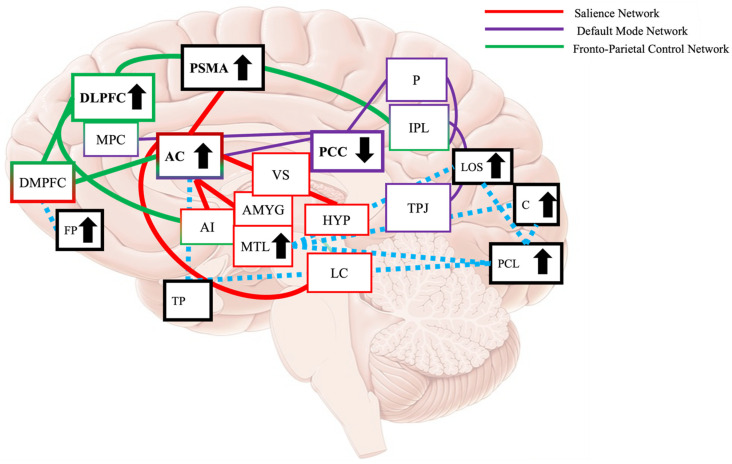
Revised model of working memory function between the DMN, FPCN, and the SN in the dementia group after music therapy. DMPFC: dorso-medial pre-frontal cortex, FP: frontal pole, DLPFC: dorsal–lateral pre-frontal cortex, MPC: middle pre-frontal cortex, AC: anterior cingulate, PSMA: pre-supplementary motor area, AI: anterior insular, MTL: medial temporal lobe, LC: locus coeruleus, TP: temporal pole, AMYG: amygdala, VS: ventral striatum, HYP: hypothalamus, PCC: posterior cingulate cortex, P: precuneus, IPL: inferior parietal lobule, TPJ: temporal parietal junction, C: cuneus, PCL: pericalcarine, LOS: the lateral occipital sulcus. The blue line shows significant connectivity differences between the C, LOS, and the PCL, between the PCL to the MTL, between the TP to the AC (caudal anterior cingulate) and to the PCL, and between the FP with the DLPFC. The black arrows in the boxes represent the significantly stronger connectivity (i.e., facing upwards) between connecting nodes. Model based on [32,36,37,67,69,163] and the results of this study. This figure is adapted from [70].

The results of this study also suggest the C, LOS, and the PCL may have been recruited by the FPCN, SN, and DMN as alternative neural pathways to facilitate working memory, or MT increased connectivity in the C, LOS, and PCL, contributing to the activation of the FPCN, SN, and DMN during the n-back after MT. Activation of alternative pathways, with evidence that music perception is still preserved in dementia (Jacobsen et al., 2015 [61]) and the compensatory action of the SN, may explain how MT can be designed to target neural networks to improve the behavioural and cognitive symptoms of dementia. Figure 8 is a model of significant connectivity differences after a cognitively intense MT program based on the results of this study, illustrating how the FPCN, the SN, the DMN, and the C, LOS, and the PCL (and other areas of significance) interact.

This study has several limitations that need to be considered. Firstly the small sample sizes of the dementia and control groups in this study were limited by attrition, funding, and the COVID-19 pandemic (challenges common in dementia research [164,165]) and consequently limited statistical power, comparisons, and interpretations of the results. In an attempt to mitigate this, case study approaches were completed for the SMMSE and n-back measures using the RCI, and the neural connectivity analysis assessed individual connectivity differences between the MEG scans before and after MT. However, we recognise caution should be taken when interpreting these results of this study, as this work needs to be replicated and extended to properly test the proposed hypotheses and the model of neural iterations with a larger number of participants and a matched clinical control group.

Another limitation that should be considered when interpreting the results of this study are potential practice effect when participants repeated the n-back for a second time. Practice effects can still occur in pre-clinical AD or amnesic MCI cohorts on trained tasks and procedural tasks in AD cohorts, particularly if the task is too simple and shows rapid learning [166]. The n-back task was designed according to guidelines to mitigate practice effects when designing cognitive tasks for dementia research [166], which included pseudorandomised trial order for each participant, and each time the n-back was completed, and a shortened (one condition) and modified practice session of the n-back task before data was recorded. However, given there is some evidence in mild dementia that practice effects occur, practice effects cannot be discounted. Future research should further consider controlling for practice effects in dementia research (e.g., letters instead of number for a second completion of an n-back task).

The variation and interpretation of the SMMSE scores and connectivity patterns were further complicated by different subtypes of dementia in this study. Empirical evidence does suggest AD and vascular dementia have similar cognitive impairments [167,168], have similar rates of cognitive decline [169], and show cognitive improvements with reminiscence-based interventions [170]. However, further research measuring the effectiveness of interventions on dementia symptoms should include participants with only a sub-type of dementia to extend understandings of how music therapy improves cognition in different sub-types of dementia and whether therapies need to be tailored to different dementia sub-types. Furthermore, we cannot rule out completely that the results of the n-back task and the significant connectivity differences after music therapy in the dementia group may have been attributed to other factors (e.g., medication profiles, practice effects, socialisation, and the physical elements of musical playing) and the possible influence of pharmaceutical treatments, and detailed use of hearing and visual aids. Lastly, there are inconsistencies between the RCI findings for the n-back task and the connectivity findings, as significance was not consistent within participants. This finding is not supported in the literature, as symptoms of dementia are usually presents with neural network dysfunction [171]. There is neuroimaging evidence suggesting potential benefits and responses to cognitive stimulation vary in people with dementia due factors such as cognitive reserve, functional status, and performance in executive functioning [172], but future research should look into the relationships between behaviour and connectivity differences after MT in dementia. It is acknowledged that these limitations are important and have implications for the interpretations of these findings, and, although we argue the MT was designed to improve common working memory and executive functional symptoms of dementia, more work is needed to establish these hypotheses.

Leading from the limitations of this study, there are several recommendations for future research. It is important to assess whether changes in neural connectivity, and executive functioning after an MT program persist in any degree after the intervention for people living with dementia. Currently, longitudinal improvements from music therapy are evident [173] but are not well established [174]. Future work should measure outcomes of executive functioning and changes to functional connectivity after MT interventions in dementia at intervals after the completion of an MT program to determine a dosage to maintain changes or if long-term engagement in MT is needed.

Although fidelity checks were discussed in this study, they were not recorded, and established protocols have been published since this study has finished. Therefore, we suggest future research assessing the effectiveness of music therapy in dementia care settings should implement fidelity checks (e.g., Clinical Practice Model for Persons with Dementia [175]) to ensure the MT program is consistent across sessions and addressing the interventions it is designed for.

Future research in this field should look to identify the therapeutic mechanism by which music therapy works for improving symptoms of executive functioning and functional connectivity in dementia. This is a gap in the literature, as studies have used music therapy combined with other therapeutic interventions [176,177] clouding the exact benefit of MT on symptoms. Therefore, future research should include an active control such as an exercise or a passive music therapy group to determine the therapeutic effect.

Finally, future research should look to determine the neural mechanisms of how music therapy might improve the executive functioning for people with dementia, as this would improve therapeutic targets and designing of MT interventions for specific symptoms.

## 5. Conclusions

This study used a cognitively intense MT that focused on stimulating compensatory neural networks to improve working memory and executive functioning symptoms commonly experienced by people with dementia. There is some evidence MT may be designed to target specific cognitive process rather than global cognition, as there were trending indications that the dementia group showed positive change on all three conditions of the n-back task after MT. The intra- and inter-connectivity findings, although cautionary, suggest an interplay between the FPCN, SN, and DMN of compensatory oscillatory activity that suggested working memory improvements in the dementia group. The analysis of connectivity differences between before and after the MT also suggests the FP, TP, PCL, LOS, and C were recruited by the FPCN, SN, and DMN to maintain cognitive performance on the n-back task or were activated by the MT.

The limitations of this study do not allow conclusive interpretations to be made about the results and the effectiveness of MT for improving executing impairments experienced by people with dementia, and this study should be viewed as a study testing a hypothesis for how music therapy may work in dementia. It is, therefore, recommend that additional neuroimaging research is needed to determine how music therapy improves cognition in dementia. Overall, our study presents preliminary evidence that music therapy can engage large-scale neural networks by targeting cognitive processes and promotes activity in more distantly connected areas that support neural network activity to maintain or improve cognitive functioning in dementia. This research provides a line of future research complementing the effectiveness of music therapy for treating symptoms of dementia by showing that neural changes can occur in response to music therapy, offering some hints about the mechanisms by which music therapy may be beneficial to people with dementia.

## Figures and Tables

**Table 1:** Gender, mean age, and standard deviation of participants.

	Gender		M	SD
	M	F		
Dementia Total	2	4	86.3	6.62
AD	1	1		
Vascular Dementia	1			
Dementia Unknown		3		
Completed MEG scans				
Dementia Total	2	2	83.5	6.35
AD	1	1		
Vascular Dementia	1			
Dementia Unknown		1		
Controls	2	1	83	5

Note: M = male, F = female, SD = standard deviation, AD = Alzheimer’s disease.

**Table 2:** Average correct, incorrect and missed N-back scores for each condition and grand average for the dementia and control groups.

Dementia	0 Back		1 Back		2 Back		Total M	
	M	SD	M	SD	M	SD	M	SD
Before Therapy								
Correct	39	28.1	59	25.7	24	16.8	122	49.9
Incorrect	20	25.2	7	6.83	22	24.1	50	24.4
Missed	34	27.5	11	10.2	55	17.1	111	25.7
After Therapy								
Correct	62	19.2	62	25.2	17	21.7	141	49.4
Incorrect	4	5.66	7	11.2	9	10.5	20	26.7
Missed	17	18.9	18	25.5	62	21.7	97	49.6
Controls								
Correct	74	4.72	70	8.96	25	11.84	169	14.04
Incorrect	8	9.71	17	23.86	9	6.81	34	30.35
Missed	6	4.72	9	9.24	43	11.72	24	6.11

Note: M = mean, SD = standard deviation. Correct = the average number of correctly identified target stimuli. Incorrect = the average number button presses not associated with target stimuli. Missed = the average number of target stimuli not responded too.

**Table 3:** *t*-tests within the dementia group and between the dementia and control groups for the N-back task.

Before Music Therapy		After Music Therapy	*t*	df	*p*	*W*	*pW*	*Z*	*pZ*	Effect Size (d’)	CI	
											Lower	Upper
N-Back Task												
Overall Correct	-	Overall Correct	−1.375	3	0.263	0.859	0.256	2	0.375	−0.624	−1.758	0.464
Overall Incorrect	-	Overall Incorrect	1.701	3	0.187	0.894	0.402	9	0.250	0.850	−0.38	1.982
Overall Missed	-	Overall Missed	0.640	3	0.566	0.851	0.228	6	0.875	0.32	−0.712	1.308
Dementia		Controls										
Before Music Therapy												
Overall Correct	-	Overall Correct	−1.409	2	0.294	0.998	0.919	0.998	0.919	−0.813	−2.106	0.596
Overall Incorrect	-	Overall Incorrect	0.652	2	0.596	0.928	0.481	0.982	0.745	3.396	0.233	6.683
Overall Missed	-	Overall Missed	5.882	2	0.0028	0.982	0.745	0.829	0.185	0.361	−0.854	1.502
After Music Therapy												
Overall Correct	-	Overall Correct	−0.537	2	0.645	0.949	0.567	0.928	0.481	−0.31	−1.443	0.889
Overall Incorrect	-	Overall Incorrect	−1.519	2	0.268	0.829	0.185	0.949	0.567	−0.876	−2.202	0.566
Overall Missed	-	Overall Missed	2.834	2	0.105	1.000	1.000	1	1	1.63	−0.260	3.459

Note: df = degrees of freedom, *W* = Shapiro Wilk Statistic, *pW* = Shapiro Wilk Statistic at alpha at 0.05, *Z* = Wilcoxon Signed Rank Test statistic, *pZ* = Wilcoxon Signed Rank Test statistic alpha at 0.05, Effect Size = Cohens d’ statistic, CI = Confidence Interval.

**Table 4:** Number (%) of correct and missed (target present) and incorrect (distractor) trials by N-back conditions.

	2-Back				1-Back				0-Back			
	Correct (%)	Incorrect (%)	Missed (%)	d′	Correct (%)	Incorrect (%)	Missed (%)	d′	Correct (%)	Incorrect (%)	Missed (%)	d′
Dementia Pre-intervention												
1	19 (23.8)	2 (1.7)	60 (75.0)	1.41	76 (95.0)	2 (1.7)	4 (5.00)	3.77	20 (25.00)	9 (7.5)	60 (75.00)	0.77
2	37 (46.3)	54 (45.0)	41 (51.3)	0.03	77 (96.3)	8 (6.7)	1 (1.25)	3.28	80 (100)	8 (6.67)	0 (0.00)	3.74
3	2 (2.5)	5 (4.2)	77 (96.3)	−0.23	60 (75.0)	0 (0.0)	20 (25.00)	3.82	21 (26.25)	13 (10.83)	58 (72.50)	0.61
4	37 (46.3)	27 (2.5)	42 (52.5)	0.66	22 (27.5)	16 (13.3)	56 (70.00)	0.51	36 (45.00)	6 (5.00)	43 (53.75)	1.53
Dementia Post-intervention												
1	1 (1.3)	1 (0.8)	78 (97.5)	0.15	76 (95.0)	3 (2.5)	4 (5.00)	3.60	79 (98.75)	0 (0.00)	1 (1.25)	5.39
2	48 (60.0)	7 (5.8)	31 (38.8)	1.82	73 (91.3)	1 (0.8)	6 (7.50)	3.75	78 (97.50)	0 (0.00)	1 (1.25)	5.10
3	3 (3.8)	3 (2.5)	76 (95.0)	0.18	74 (92.5)	1 (0.8)	5 (6.25)	3.83	48 (60.00)	4 (3.33)	31 (38.75)	2.09
4	15 (18.8)	24 (20.0)	64 (80.0)	−0.05	24 (30.0)	24 (20.0)	56 (70.0)	0.32	43 (53.75)	12 (10.00)	36 (45.00)	1.38
Control												
1	11 (13.8)	14 (11.7)	68 (85.0)	0.10	76 (95.0)	6 (5.0)	4 (5.00)	3.29	69 (86.25)	19 (15.83)	11 (13.75)	2.09
2	31 (38.8)	11 (9.7)	48 (60.0)	1.04	75 (93.8)	44 (36.7)	4 (5.00)	1.87	78 (97.5)	6 (5.00)	2 (2.50)	3.60
3	32 (40.0)	1 (0.8)	52 (65.0)	2.14	60 (75.0)	0 (0.0)	20 (25.00)	3.82	76 (95.00)	0 (0.00)	4 (5.00)	4.79

Note: To deal with floor and ceiling effects, 0.25 was subtracted (added) from (to) 100% correct (incorrect) responses before d′ was calculated. Target trials = 80; distractor trials = 120.

**Table 5:** Reliable change index statistics for the SMMSE and N-back task for dementia participants.

Participant	SMMSE										
1	−5.64										
2	2.41										
3	−6.44										
4	0										
5	2.41										
6	−0.81										
N-Back Task											
Participant	Correct				Incorrect				Missed		
	0	1	2	Total	0	1	2	Total	0	1	2
2	4.96	2.57	0.18	7.71	−9.92	0.18	−0.36	−10.10	−4.53	−2.61	−0.17
3	1.28	0.36	−4.04	−1.8	1.10	1.47	−0.73	1.83	−2.02	6.61	4.04
4	10.84	0	−3.31	7.53	−1.65	−0.18	−0.18	−2.01	−11.76	0	3.30
5	−0.36	−0.72	2.20	0.91	−1.47	−1.28	−8.63	−11.39	0.18	0.91	−1.81

Note: SMMSE = Standardised Mini Mental State Examination.

**Table 6:** Connectivity plots that meet FDR threshold of significance of *p* < 0.05.

Participant	Frequency Band			
	Theta	Alpha	Beta	Gamma
2	FDR	*p*-value	FDR	FDR
3	*p*-value	*p*-value	FDR	FDR
4	FDR	FDR	*p*-value	FDR
5	*p*-value	*p*-value	*p*-value	FDR

Note: FDR = connectivity analysis that survived the false discovery rate test at *p* < 0.05, *p*-value = the connectivity analyses that did not survive the FDR test and were plotted using the *p*-values at *p* < 0.05.

## Data Availability

The raw data supporting the conclusions of this article will be made available by the authors on request.

## Supplementary Materials

The following supporting information can be downloaded at: https://www.mdpi.com/article/10.3390/biomedicines14020452/s1. Figure S1. Visual of the Music Therapy Procedure at Each Week of the Program. Figure S2. Additional Steps of the MEG Analysis Used in This Study.
