# Supplementary material for: Can Music Therapy Improve Cognition in Dementia as Measured with Magnetoencephalography: A Hypothesis Study
Source: Biomedicines. 2026 Feb 17;14(2):452. doi: 10.3390/biomedicines14020452 (PMC12937690; doi:10.3390/biomedicines14020452)
Supplement: Supplementary file 1 [file biomedicines-14-00452-s001.zip › biomedicines-4111491-supplementary.pdf]

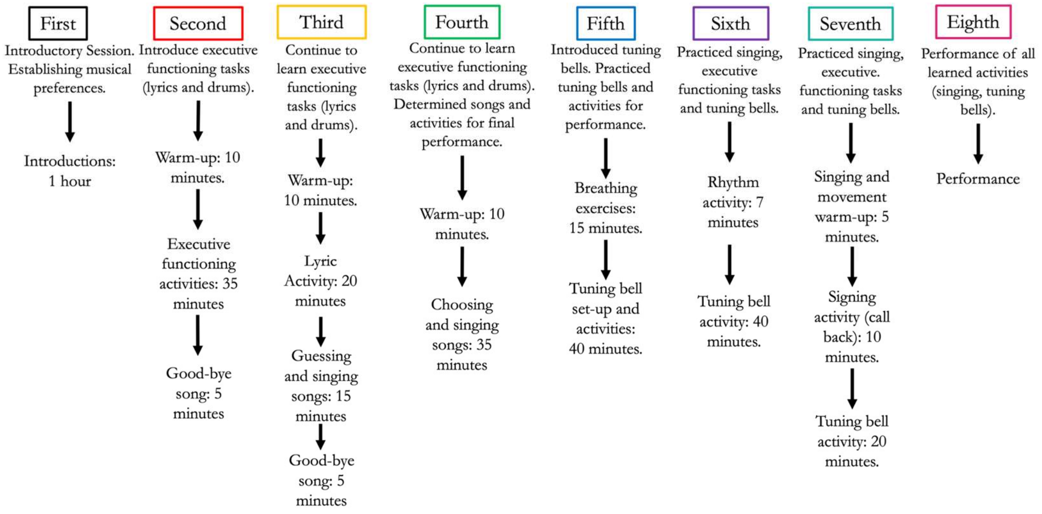

Figure S1. Visual of the Music Therapy Procedure at Each Week of the Program.

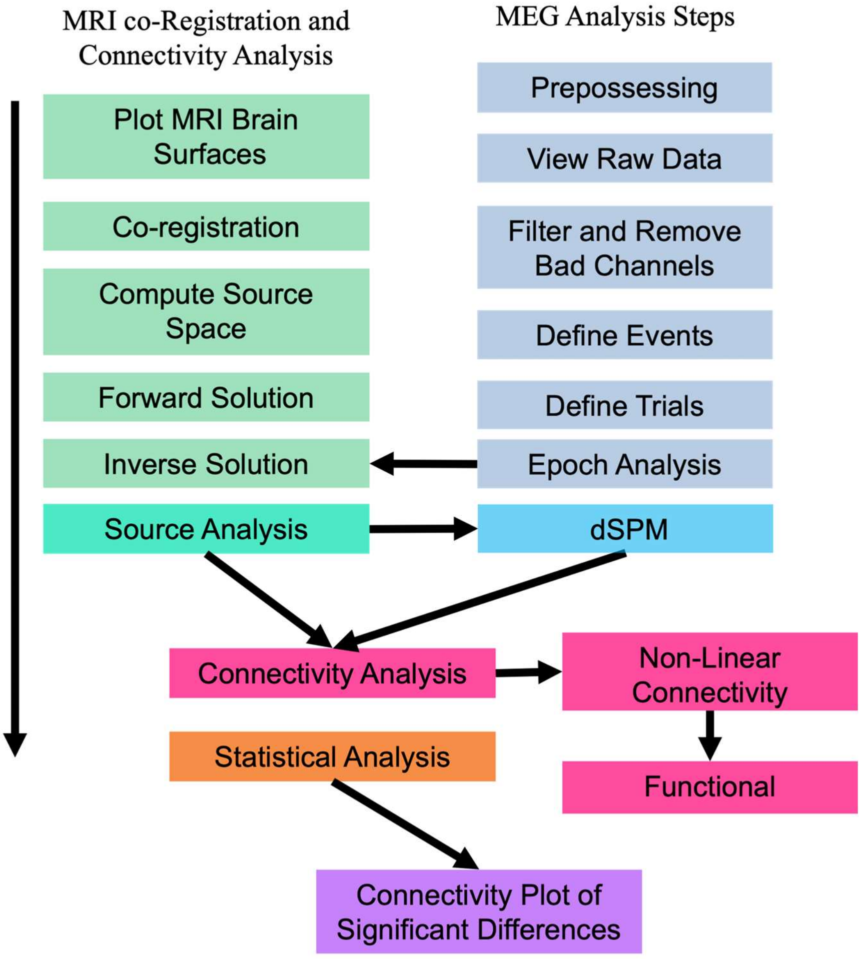

Figure S2. Additional Steps of the MEG Analysis Used in This Study.
